# The NRON complex controls circadian clock function through regulated PER and CRY nuclear translocation

**DOI:** 10.1038/s41598-019-48341-8

**Published:** 2019-08-15

**Authors:** Yool Lee, Yang Shen, Lauren J. Francey, Chidambaram Ramanathan, Amita Sehgal, Andrew C. Liu, John B. Hogenesch

**Affiliations:** 1 Department of Systems Pharmacology and Translational Therapeutics, University of Pennsylvania Perelman School of Medicine, Philadelphia, PA 19104 USA; 2 Penn Chronobiology, Howard Hughes Medical Institute, Department of Neuroscience, Perelman School of Medicine, University of Pennsylvania, Philadelphia, PA 19104 USA; 3 Department of Physiology and Functional Genomics, University of Florida College of Medicine, Gainesville, FL 32610 USA; 4 Department of Biological Sciences, University of Memphis, Memphis, TN 38152 USA; 5 Divisions of Human Genetics and Immunobiology, Cincinnati Children’s Hospital Medical Center, Cincinnati, OH 45229 USA

**Keywords:** Circadian rhythms, RNAi

## Abstract

Post-translational regulation plays a central role in the circadian clock mechanism. However, nucleocytoplasmic translocation of core clock proteins, a key step in circadian timekeeping, is not fully understood. Earlier we found that the NRON scaffolding complex regulates nuclear translocation of NFAT and its signaling. Here, we show that components of the NRON complex also regulate the circadian clock. In peripheral cell clock models, genetic perturbation of the NRON complex affects PER and CRY protein nuclear translocation, dampens amplitude, and alters period length. Further, we show small molecules targeting the NFAT pathway alter nuclear translocation of PER and CRY proteins and impact circadian rhythms in peripheral cells and tissue explants of the master clock in the suprachiasmatic nucleus. Taken together, these studies highlight a key role for the NRON complex in regulating PER/CRY subcellular localization and circadian timekeeping.

## Introduction

Most organisms evolved endogenous circadian clocks that control ~24 h rhythms in physiology. These endogenous clocks allow organisms to anticipate and adapt to cyclic environmental changes and are critical for fine tuning physiology. In humans, disruption of the circadian system is associated with a variety of diseases, including sleep disorders, metabolic syndrome, cardiovascular diseases, and cancer^1–4^. The molecular clock is a transcriptional/translational feedback loop that generates rhythmic gene expression output. In this loop, the bHLH-PAS domain-containing transactivators, BMAL1 and CLOCK (or its paralog, NPAS2), bind as a heterodimeric complex to E-box sequences in the promoters of Period (*PER1*, *2*, *3*) and Cryptochrome (*CRY1*, *2*) genes, activating their expression. The PER and CRY protein products form their own complex, and, upon translocation to the nucleus, repress BMAL1/CLOCK^4–6^. Transcription factors of at least two other transcriptional feedback loops (i.e. D-box and RORE) regulate components of this E-box loop to impart robustness and regulate circadian output^7–9^.

While the core clock mechanism is accepted, critical gaps exist. For example, the repression step is thought to involve: PER/CRY protein accumulation, modification, nuclear transport, repression of BMAL1/CLOCK in the nucleus, and eventual degradation. Previous studies identified several key factors, including CSNK1D/E^10–12^, GSK3B^13,14^, PP1, PP2A^14–16^, FBXL3 and FBXL21^17–19^, and KPNB1^20^, that function to modify PER and CRY proteins to control their abundance and activities (reviewed in^21,22^). While it is generally accepted that these factors regulate the nuclear translocation and accumulation the PER/CRY repressor complex, the precise steps in which they do so remain poorly understood.

**Figure 1:**
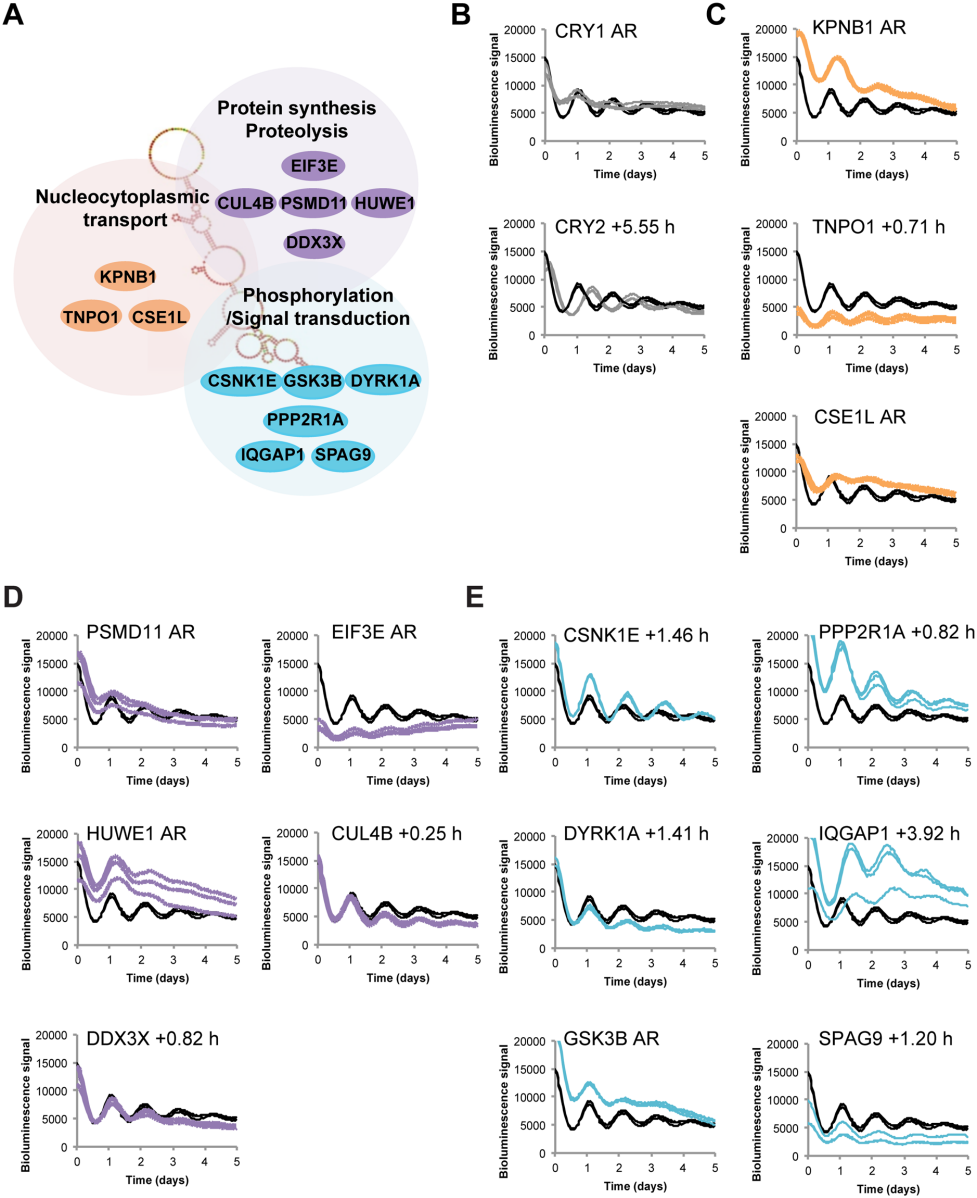
RNAi knockdown of the NRON complex components alters circadian oscillations in U2OS cells. (**A**) Schematic illustration of NFAT signaling complex components. (**B**–**E**) U2OS cells stably expressing the *Bmal1* promoter-driven destabilized luciferase (*pBmal1*-dLuc) were reverse transfected with targeted siRNAs, synchronized with dexamethasone (dex) 24 h post-transfection and bioluminescence was monitored in real-time at a 1 hr sampling resolution over 6 days. Data are mean ± S.E. (n = 3 biological replicates). (**B**) RNAi knockdown effects of negative (Neg si) and positive controls (*CRY1*, *CRY2*). **(C)** RNAi knockdown effects of nuclear transport components (*KPNB1*, *CSE1L*, *TNPO1*). *KPNB1* and *CSE1L* displays arrhythmic (AR) phenotype when depleted. **(D)** RNAi knockdown effects of protein synthesis and degradation components (*PSMD11*, *EIF3E*, *HUWE1*, *CUL4B*, *DDX3X*). (**E**) RNAi knockdown effects of signal transduction pathway components (*CSNK1E*, *GSK3B*, *DYRK1A*, *PPP2R1A*, *IQGAP1*, *SPAG9*).

Earlier, we identified a long non-coding RNA (lncRNA), named NRON, the noncoding repressor of NFAT (nuclear factor of activated T cells)^23^. NFAT is a family of transcription factors regulated by Ca^2+^ to control gene expression. NFAT signaling contributes to diverse processes, including the nervous system, the immune and inflammatory responses, neurodegeneration, and cancer (reviewed in^23–26^). NRON plays a key role in the assembly of a large RNA/protein complex, consisting of three distinct, functionally interrelated groups of protein components, namely the NRON complex^23,27^ (Fig. 1A): (1) kinases (CSNK1E, GSK3B, DYRK1A), phosphatases (PP2A, calcineurin (or PP2B), PPP2R1A), and scaffolding proteins (IQGAP1) are involved in signal transduction and post-translational modifications; (2) EIF3E, CUL4B, PSMD11, and HUWE1, of which PSMD11 and CUL4B are proteasome components, regulate protein synthesis and turnover; and (3) CSE1L, KPNB1 and TNPO1 participate in nucleocytoplasmic transport. The NRON complex regulates NFAT signaling at the critical step of subcellular localization^23,25,27^: under basal conditions, NFAT is hyper-phosphorylated, cytosolic and unstable; upon pathway activation, the Ca^2+^ sensor calmodulin (CaM) activates the serine/threonine phosphatase calcineurin (CaN, aka PP2B), which then dephosphorylates NFAT; then the hypophosphorylated NFAT is freed to translocate to the nucleus to induce target gene expression. As such, CaN serves as a master repressor of NFAT nuclear translocation and transcriptional activity^25,26^.

Intriguingly, we found that the same complex that regulates NFAT also regulates translocation of clock proteins PER and CRY^20^. More specifically, the kinases in the complex (CSNK1E, GSK3B, DYRK1A) were shown to regulate NFAT function^10,27,28^. These kinases also regulate phosphorylation-related proteolysis and nuclear entry of PER and CRY proteins^13,14,20,22^. Further, two other components of the NRON complex, nuclear importin beta (KPNB1) and nuclear import carrier Transportin 1 (TNPO1) were recently shown to play a key role in post-translational regulation of the PER/CRY complex^20,29^. Thus, the NFAT and circadian clock pathways share functional components.

Here, we extend these studies to other components of the NRON complex and characterize their role in nucleocytoplasmic shuttling of the PER/CRY complex. We demonstrate that these regulations are functional using both peripheral clock models and *ex vivo* suprachiasmatic nucleus (SCN) explants. Collectively, these studies point to the role of the NRON complex as a flexible module regulating nucleocytoplasmic shuttling of at least two critical signal transduction pathways, NFAT, and the circadian clock.

## Results

### Knockdown of the NRON complex components alters circadian rhythms in human and mouse cellular clock models

Previous studies including ours show that several components of the NRON complex, including CSNK1E, GSK3B, and DYRK1A, regulate clock function in mammals^13,21,22^. Several components and their analogs have knockdown phenotypes in our previous siRNA genomic screen, including *PSMD11*, *PSMD13*, *DDX3X*, *DDX56*, *KPNB1*, and *KPNA4*^30^. We showed that importin-beta (KPNB1) directly interacts with PER2 and mediates PER/CRY nuclear translocation, and in so doing, regulates circadian rhythms in human cells and in flies^20^. As these NRON complex components perform independent but interrelated functions in regulating nucleocytoplasmic protein abundance and activities, we hypothesized that other members of the complex also play roles in regulating clock function. To test this, we used RNA interference (RNAi) to knock down the complex components individually in human U2OS cells stably expressing the *Bmal1* promoter-driven luciferase reporter and performed kinetic bioluminescence assays to assess clock function. We show that knockdown of 12 of 14 genes altered circadian rhythms in these cells (Fig. 1B–E), without adversely impacting cell viability (Fig. S1A). For example, knockdown of nuclear import and export components, *KPNB1* and *CSE1L*, caused arrhythmicity, consistent with our previous findings^20^. Three genes involved in protein synthesis and proteolysis (*EIF3E*, *PSMD11*, *HUWE1*) led to arrhythmicity upon knockdown. Similar results were obtained from an independent mouse MMH-D3 hepatocyte clock model^31^ (Fig. S1B). The majority of these genes that are involved in phosphorylation and signal transduction caused long period phenotypes upon knockdown, including *DYRK1A*. It is noted that the *DYRK1A* knockdown phenotype is consistent with our previous genome-wide screen in the U2OS cell model^30^, but different from a previous report that showed a short period length phenotype in a fibroblast cell model^13^. We confirmed the long period phenotype in U2OS cells using Harmine, a specific DYRK1A inhibitor^32^ (Fig. S1C), which is also consistent with a previous study showing a similar phenotype in Per2::Luc embryonic fibroblasts and the SCN^33^. This phenotypic difference may reflect cell line or type-specific clock function. Taken together, these results suggest that the NRON complex, known for its role in regulating NFAT signaling, also regulates the circadian clock.

### Components of the NRON complex interact with core clock proteins

**Figure 2:**
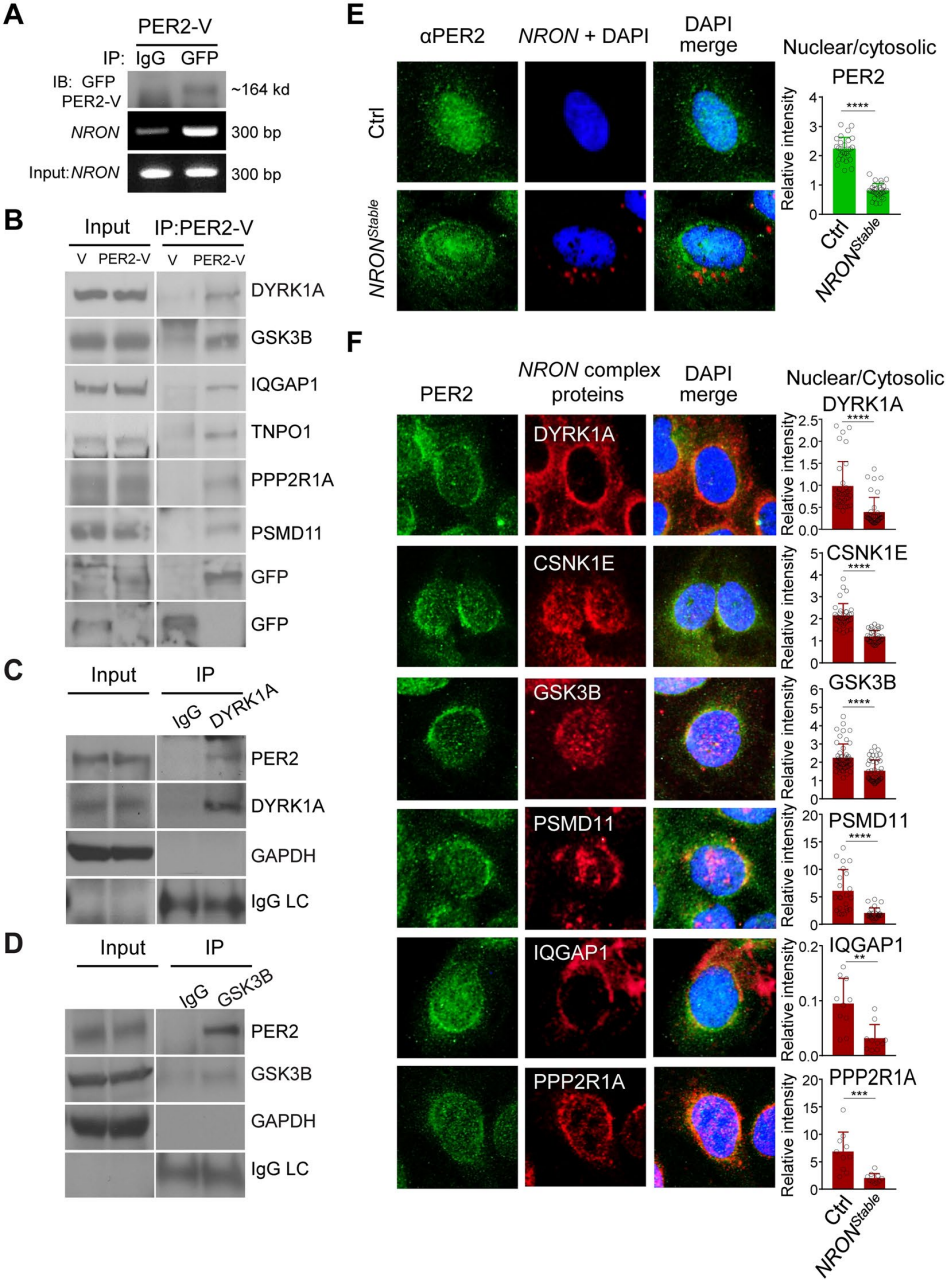
Biochemical interaction and colocalization of the PER/CRY complex with the *NRON* complex. **(A)** RNA-binding protein immunoprecipitation (RIP) assay detected the *NRON* association with PER2. PER2 and *NRON* in the immunoprecipitated protein/RNA complex were determined by immunoblot analysis and quantitative RT-PCR, respectively. PER2-V: PER2 fused with Venus, an enhanced variant of GFP. (**B**) Co-immunoprecipitation analysis to detect PER2 interactions with the NRON complex components in human U2OS cells. Cell lysates from transfected cells were immunoprecipitated with anti-GFP antibody to capture Venus (V) or PER2-Venus (PER2-V), followed by immunoblotting with the indicated antibodies. The original full immunoblots are presented in Fig. S2. **(C**,**D)** Co-immunoprecipitation of PER2 with endogenous DYRK1A and GSK3B in the liver. Liver extracts were immunoprecipitated and then immunoblotted with the indicated antibodies. Antibodies against GAPDH and IgG light chain (IgG LC) serve as controls. The original full immunoblots are presented in Fig. S2. (**E**) Combined RNA fluorescent *in situ* hybridization (FISH) and immunofluorescence (IF) analysis of subcellular colocalization of *NRON* and PER2 in control cells (CTL) and cells stably expressing *NRON* (*NRON*^*Stable*^). The nuclear/cytoplasmic ratio of PER2 in control (Ctrl) and *NRON*^*Stable*^ cells is shown as a scatter plot (right). p**** < 0.00001 by one way ANOVA followed by Dunnett’s multiple-comparisons test. Data are presented as mean ± SD (n = 27~30). Fluorescence microscopy filter sets: FITC (green, PER2), TRITC (red, *NRON*), and DAPI (blue, nuclei). **(F)** Immunofluorescence imaging analysis to detect subcellular colocalization of PER2 with the NRON complex proteins. U2OS cells stably expressing *NRON* (*NRON*^*Stable*^) were fixed and immunostained with antibodies against PER2 and other proteins as indicated. The nuclear/cytoplasmic ratio of the NRON complex proteins in control (Ctrl) and *NRON*^*Stable*^ cells (*NRON*^*stable*^) is shown (right). ****p < 0.00001, ***p < 0.001, **p < 0.01 by one way ANOVA followed by Dunnett’s multiple-comparisons test. Data are presented as mean ± SD (n = 10~38). Fluorescence microscopy filter sets: FITC (green, PER2), TRITC (red, individual NRON components), and DAPI (blue; nuclei). DAPI merged images show colocalization.

CSNK1E, GSK3B, and DYRK1A, three kinase components of the NRON complex, are known to be involved in phosphorylation-dependent stability and nuclear localization of PER and CRY in both flies and mammals^21,22^. To further investigate the direct role of the NRON complex, we examined the interactions of the complex components with core clock proteins. Our recent study showed that KPNB1, which directs protein nuclear trafficking, directly interacts with PER and CRY. Perturbation of KPNB1 has strong effects on PER2 nuclear translocation and PER-CRY complex formation^20^. Previous studies also characterized the interaction between CSNK1E and PER2^11,34^. Using RNA-protein immunoprecipitation (RIP) and quantitative PCR analyses, we show that Venus-tagged PER2 (PER2-V) associated with *NRON* in U2OS cells (Figs 2A and S2). Co-immunoprecipitation assays detected PER2-V interactions in transfected U2OS cells with endogenous clock regulatory kinases DYRK1A and GSK3B, as well as other complex components including IQGAP1 (CaM-binding scaffolding protein), TNPO1 (nuclear import protein), PPP2R1A (protein phosphatase regulatory subunit), and PSMD11 (a 19S proteasome lid component) (Fig. 2B). Importantly, we also detected endogenous PER2 interactions with DYRK1A and GSK3B in the mouse liver (Fig. 2C,D).

If PER2 interacts with the NRON complex, they must co-localize in the same subcellular compartment. Using immunofluorescence imaging combined with RNA fluorescence *in situ* hybridization (FISH), we show that PER2 colocalizes with NRON and the NRON complex components (DYRK1A, CSNK1E, GSK3B, IQGAP1, PPP2R1A, and PSMD11) at the perinuclear membrane in U2OS cells stably expressing *NRON* (Figs 2E,F and S3). Notably, *NRON* reduced the abundance of PER2 in the nucleus and the other complex components. These data suggest that PER2 associates with the NRON complex components and colocalizes at the perinuclear region.

### NRON knockdown alters the status of core clock proteins and clock function

**Figure 3:**
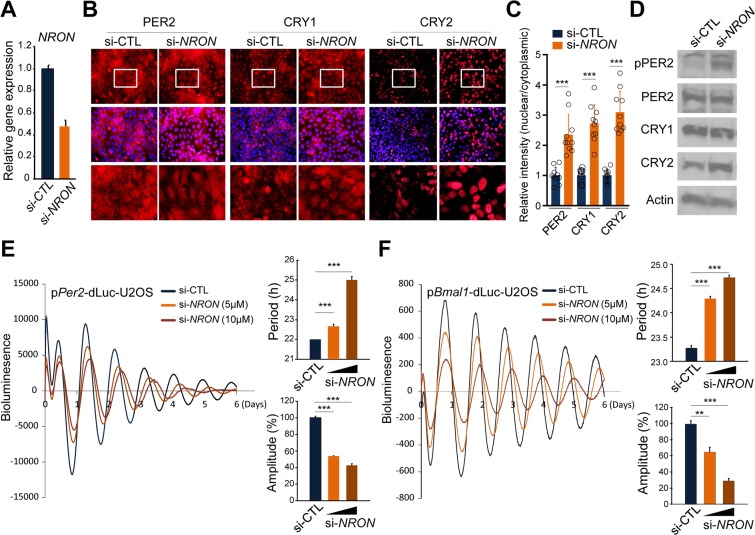
NRON knockdown enhances nuclear localization of PERs/CRYs and dampens circadian gene expression. **(A)** Quantitative RT-PCR analysis of *NRON* knockdown efficiency in control (si-CTL) and *NRON* siRNA-treated U2OS cells (si-*NRON*). Data are mean ± S.E. (n = triplicate samples). **(B)** siRNA-mediated *NRON* knockdown effect on subcellular localization of endogenous PER2, CRY1, and CRY2 in U2OS cells. Upper panel: staining of PER2, CRY1 and CRY2. Middle: Dapi-merged. Low panel: higher magnification of selected rectangular boxes. **(C)** Quantitative analysis of the nuclear/cytoplasmic ratio of PER2, CRY1, and CRY2 in control (Ctrl; dark blue) and *NRON*-depleted (si-*NRON*; orange) cells are shown with scatter plots ***p < 0.0001 by one way ANOVA followed by Dunnett’s multiple-comparisons test. Data are presented as mean ± SD (n = 10). **(D)** Western blot analysis of *NRON* knock-down effect on phosphorylation and abundance of PER/CRY proteins using the specific antibodies as indicated. pPER, anti-triphospho-PER2 antibody (Ser662/Ser665/Ser668). Actin is loading control. **(E**,**F)** Normalized bioluminescence recordings of dexamethasone-synchronized U2 OS cells expressing Per2 or *Bmal1* promoter-driven destabilized luciferase (*pPer2*-dLuc, *pBmal1*-dLuc) transfected with control or increasing dose of *NRON* (0.5 μM, 1 μM) siRNA-treated cells. Mean relative periods (right upper) and amplitudes (right lower) ± SD (n = 3) are shown (***p < 0.005, by two-tailed student t-test).

Stable expression of NRON in U2OS cells affected the subcellular localization of PER2, as well as other NRON complex components. Next, we examined the effects of *NRON* loss of function on core clock proteins and clock function. In contrast to the effect of *NRON* overexpression, RNAi knockdown significantly increased nuclear accumulation of endogenous PER2, CRY1 and CRY2 (Fig. 3A–C). *NRON* knockdown also substantially increased PER2 phosphorylation without an obvious effect on its overall abundance, whereas CRY2 levels, but not CRY1, were increased (Fig. 3D). Further, compared to a non-specific control siRNA, *NRON* knockdown caused significantly longer period length and reduced rhythm amplitude in a dose-dependent manner (Fig. 3E,F). This clock phenotype is consistent with the increased abundance of nuclear PER and CRY repressors.

### Perturbation of Ca^2+^ signaling alters clock function in U2OS cells

**Figure 4:**
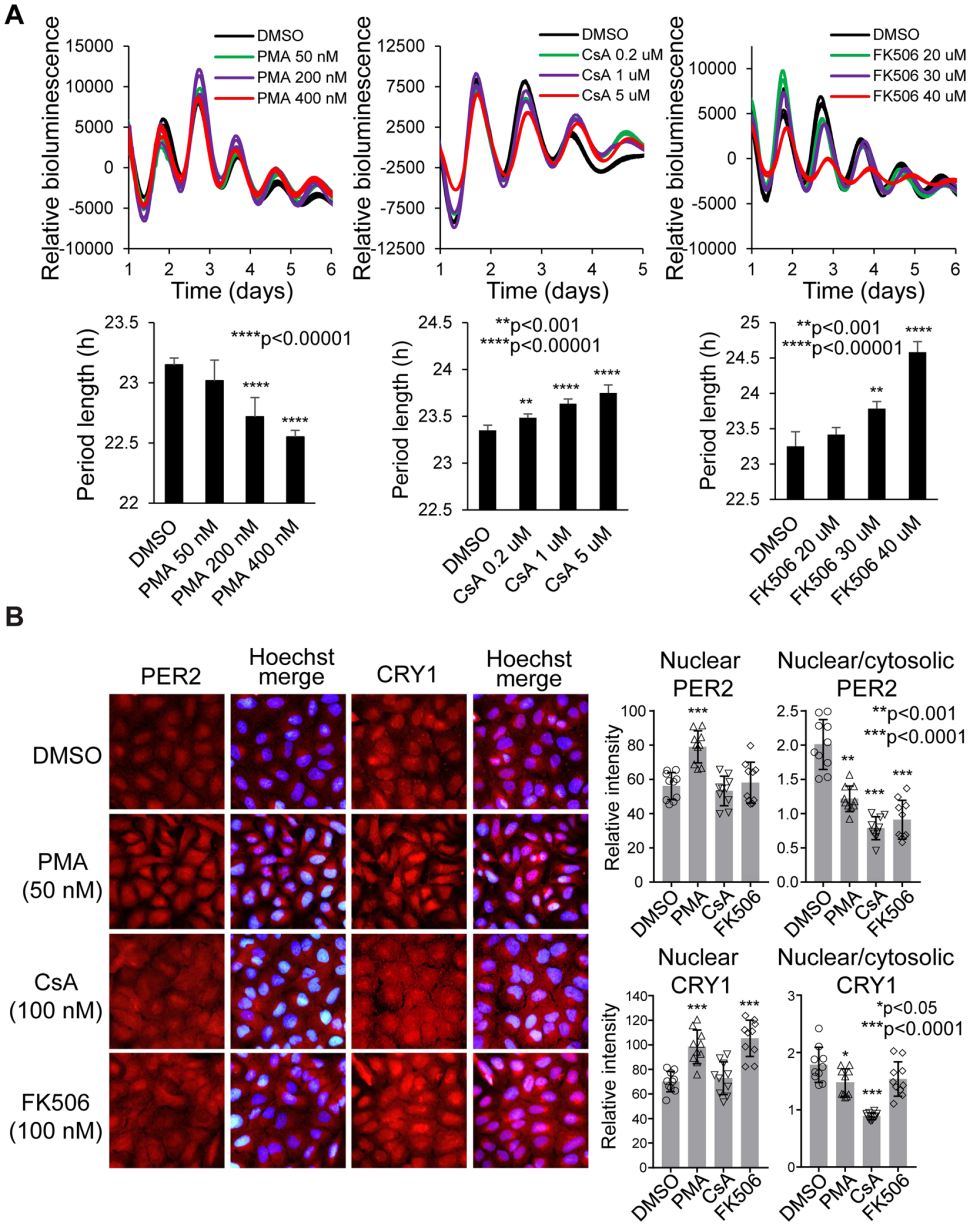
Pharmacological perturbation of the NRON pathway altered PER/CRY nuclear transport and circadian rhythms in U2OS cells. **(A)** Representative normalized bioluminescence records of circadian rhythms in U2OS cells expressing the *Bmal1*-dLuc reporter. CaN inhibitors CsA (10 μM) and FK506 (30 μM) caused long period length, consistent with RNAi knockdown effects. **(B)** PMA activator and CsA and FK506 inhibitors have opposite effects on nuclear translocation of PER2 and CRY1. PER2 and CRY1 proteins were detected by immunostaining using anti-PER2 and anti-CRY1 antibodies. Representative images (upper) are shown with quantitative assessment of the nuclear/cytoplasmic ratio (lower) for the effect on PER2 and CRY1 nuclear abundance after 6 h of treatment. Data are presented as mean ± SD (n = 10); *p < 0.05, ***p < 0.0001 by two-tailed student’s t-test. The effect on PER1 and CRY2 are presented in Supplemental Data Fig. S5.

Our data here and previous findings suggest that the NRON complex regulates both NFAT signaling and the circadian clock^20,23,27^. NFAT is regulated by the Ca^2+^ sensor calmodulin (CaM) and the phosphatase calcineurin (CaN). CaN is a central regulator of the NFAT pathway; CaN dephosphorylates NFAT, leading to its nuclear translocation and transactivation. Therefore, we reason that this pathway might also impact the clock and that perturbation of Ca^2+^ signaling would affect cellular circadian rhythms. To test this hypothesis, we leveraged well-established pharmacological agents and cellular clock models and tested their effects on circadian rhythms in U2OS cells. Cyclosporine A (CsA) and FK506 (Tacrolimus) are classic CaN inhibitors that block NFAT dephosphorylation, trapping NFAT in the cytoplasm^35^. We show that, while phorbol 12-myristate 13-acetate (PMA), an activator of NFAT signaling, shortened the period length, both CsA and FK506 lengthened period in these cells (Figs 4A and S5A).

We asked whether genetic perturbation of CaN via RNAi knockdown also affects clock function. CaN is composed of a catalytic subunit and a regulatory subunit. There are three catalytic subunits encoded by *PPP3CA*, *PPP3CB* and *PPP3CC*. We show that, among the three subunit genes, *PPP3CC* knockdown in human U2OS cells significantly lengthened period (Fig. S4A). A similar phenotype was also observed in mouse MMH-D3 cells when *Ppp3cc* was knocked down by lentiviral shRNAs (Fig. S4B). Taken together, our data from both genetic and pharmacological perturbation suggest that the CaM-CaN axis regulates circadian clock function.

### Perturbation of Ca^2+^ signaling alters PER/CRY nuclear translocation in U2OS cells

The CsA and FK506 effect on clock function in U2OS cells raised the possibility that they affected the nuclear translocation of core clock proteins, particularly PER and CRY, as in their effect on NFAT. To test this, we performed immunofluorescence imaging to determine the effect of perturbation of Ca^2+^ signaling on subcellular localization of PER1, PER2, CRY1 and CRY2 in these cells^25^. Compared to DMSO control, PMA significantly increased the abundance of all PER and CRY repressor proteins in the nucleus (Figs 4B and S5B,C), consistent with the shortened period length in PMA treated cells (Figs 4A and S5A). However, we did not observe the opposite effects from the CsA and FK506 inhibitors. Overall, CsA and FK506 lowered the relative nuclear/cytoplasmic levels of PER and CRY repressors, but did not considerably alter nuclear PER/CRY levels. On the other hand, PMA did not increase the relative nuclear/cytoplasmic ratio, as would be expected from the CsA and FK506 effects. The PMA did increase the protein levels in the cytosol in most cells. This increase may be attributable to transcriptional induction and reduced protein degradation^36–38^. Thus, our data support a role of Ca^2+^ signaling and the CaM-CaN axis in regulating clock function, but the mechanistic details are not clear and require future studies.

### Perturbation of calcineurin and Ca^2+^ signaling alters the SCN clock function

**Figure 5:**
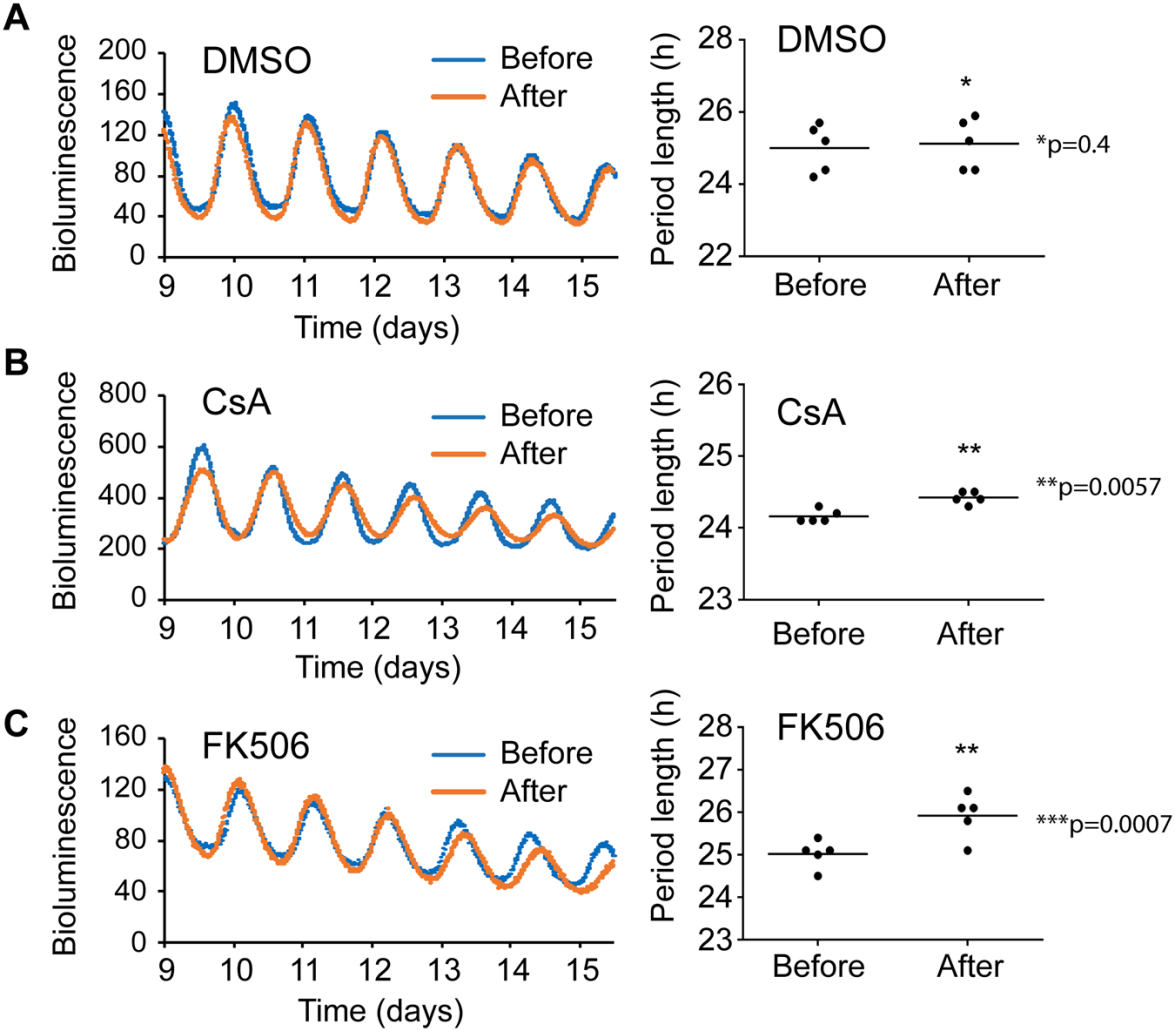
Pharmacological perturbation of the NRON pathway altered circadian rhythms in the SCN clock. **(A**–**C)** SCN explants from *Per2*^*Luc*^ reporter mice were treated with either DMSO (**A**), 20 μM CsA (**B**), or 30 μM FK506 (**C**), and the bioluminescence records before and after each treatment are plotted together for direct comparison. Representative bioluminescence records are shown on the left. Period data (right) are mean ± SD (n = 4–5 mouse SCN slices). Compared to DMSO control (25.00 hr ± 0.67 before treatment, n = 5; 25.12 hr ± 0.70 after treatment, n = 5; p = 0.4), both FK506 and CsA lengthened periods in SCN explants. In comparison, the FK506 effect (25.02 hr ± 0.33 before FK506, n = 5; 25.92 hr ± 0.52 after FK506, n = 5; p = 0.0057) was stronger than CsA (24.16 hr ± 0.09 before CsA, n = 5; 24.42 hr ± 0.08 after CsA, n = 5; p = 0.0007). Period and amplitude data (right) are mean ± SD (n = 4 mouse SCN slices). These treatments altered tissue-autonomous circadian rhythms of SCN explants cultured *ex vivo*.

Our findings that CaN and the NRON complex regulate clock function in multiple cell models (U2OS, MMH-D3), and the well recognized role of Ca^2+^ signaling in regulating SCN circadian timekeeping in the SCN^39^, suggested a ubiquitous modifier role and raised the possibility that the pathway also regulates the SCN clock. We dissected SCN slices from the PER2::LUC fusion knockin (*Per2*^*Luc*^) mice and cultured the tissue explants *ex vivo*^40,41^. Compared to DMSO control treatment (Fig. 5A), both CsA and FK506 significantly lengthened period in SCN explants (Fig. 5B,C). Taken together, our data suggest that the NRON complex regulates clock function not only in peripheral oscillators but also in the master SCN clock.

## Discussion

Circadian oscillations are cell-autonomous and self-sustaining. These properties distinguish the clock from many other cellular processes. One of the key steps in establishing circadian oscillation is the cytoplasmic to nuclear translocation of the PER/CRY repressor complex. This process occurs via PER/CRY protein degradation and gradual accumulation in the cytoplasm, followed by nuclear transport and subsequent repression of BMAL1/CLOCK activity. Several players known to modify PER/CRY play critical roles in setting the clock speed^21,22^. In particular, to maintain circadian gene transcription, clock protein abundance must be coupled with nuclear translocation. In this context, the NRON complex, which is positioned to couple protein synthesis with degradation, and cytoplasmic protein abundance with nuclear transport, provides the missing link. Our studies show that the NRON complex regulates nucleocytoplasmic partitioning of PER/CRY, and most complex members display strong knockdown phenotypes in circadian assays.

The NRON complex links enzymes that catalyze protein modifications with those that target them either for degradation or for nuclear transport. More specifically, NRON mediates the assembly of Ca^2+^ signaling scaffolding protein (IQGAP1), kinases and phosphatases (CSNK1, DYRK1A, GSK3B), proteasome components and stability factors (PSMD11, CUL4B), and nuclear import and export factors (KPNB1, TNPO1, CSE1L). This complex appears to localize at the perinuclear regions, where the nuclear transport machinery is located. This perinuclear scaffolding architecture confers regulatory specificity, efficiency, and strength^42,43^. While we have a reasonable understanding of the steady-state nuclear localization of PER/CRY, we do not have a detailed description of how their cytoplasmic degradation and nuclear translocation are linked dynamically throughout the day. In this context, it is plausible that the interrelated functions of the NRON complex coordinate to enable circadian oscillations.

As most members of the NRON complex are required not only for NFAT signaling but also for clock function, these two processes are mechanistically linked. The NRON complex regulates the NFAT pathway via the CaM-CaN axis and consequently NFAT nuclear translocation. However, it is not clear whether the NRON complex affects PER/CRY function directly through the CaM-CaN axis or indirectly through the NFAT activity. This study cannot differentiate these two mechanisms. It is possible that CaN interacts with and modifies PER/CRY, affecting their activity and clock function. In this manner, the CaN-PER/CRY pathway is independent of the CaN-NFAT pathway. The CaN inhibitors likely affect PER/CRY phosphorylation states and nuclear translocation, but the mechanistic details of this regulation are not clear. We speculate that the stoichiometric ratios of the activators (e.g. BMAL1, CLOCK) and repressors (PER1, PER2, PER3, CRY1, CRY2) in the nucleus may be responsible for period length differences provoked by activators (PMA) and inhibitors (FK506, CsA). The NRON complex may also indirectly affect the clock via NFAT’s transcriptional activity, e.g. by affecting core clock gene expression. Thus, the mechanisms of NRON function likely involve PER/CRY protein post-translational modification, stability and degradations, nucleocytoplasmic partitioning, and ultimately their transcriptional activities in the nucleus. It is conceivable that these dynamic interactions and regulatory functions work to maintain a robust clock.

Experimental evidence supports rhythmic Ca^2+^/CaM-CaN-NFAT activity in the SCN and that perturbation of the NRON complex alters both circadian *Per2*^*Luc*^ and *NFAT-RE::dLuc* rhythms. In particular, CaN subunits (including its catalytic and regulatory subunits, e.g. *Ppp3ca*, *Ppp3r1*) are highly rhythmic in the liver and modestly rhythmic in the SCN^7^. ChIP-seq analysis revealed that CaN subunits are targeted by BMAL1, PER1/2 and CRY1/2^9^. Conversely, our data suggest that the NRON-NFAT pathway impacts clock function. As NFAT plays roles in diverse processes, including immune and inflammatory responses, its circadian regulation in SCN has implications for these processes as well. Hundreds of NFAT target genes are rhythmically expressed in the SCN and liver. These genes peak immediately before and after dawn in the liver and the SCN, respectively, raising the possibility that NFAT plays a role in regulating entrainment to light and food.

Previous studies show that SCN explants of *Bmal1*^−/−^ and *Cry1*^−/−^*;Cry2*^−/−^ mice display residual quasi circadian *Per2*^*Luc*^ oscillations^44,45^. The molecular origins for these oscillations remain elusive. In light of our findings, we speculate that Ca^2+^ homeostasis, signaling through the NRON pathway, might contribute to PER/CRY nuclear transport regulation; this process impinges on the E-box to generate stochastic gene expression, which underlies weak but quasi circadian oscillations. This notion is supported by findings that GPCR signaling and intracellular Ca^2+^ activities play an essential role in enabling circadian oscillations in the SCN and in fly free-running behavior^39,46,47^. Intracellular Ca^2+^ concentrations in SCN neurons display a circadian rhythm, which is cell autonomous and dependent on the core clock mechanism, but also is reinforced by a synchronized SCN neuronal network^48,49^. Further, Ca^2+^ oscillations may also arise from rhythmic behavioral and physiological functions, including locomotor activity and feeding. It is interesting to note that the CaN-NFAT pathway, as an input to the clock, controls activity-dependent circadian gene expression in skeletal muscle^50^. This crosstalk between Ca^2+^ signaling and the circadian clock provides a mechanism where each process can reinforce or influence the other during normal or pathological conditions.

## Methods

### Ethics statement

Animals were maintained in the animal facility at the University of Florida and Cincinnati Children’s Hospital Medical Center. All animal experiments were conducted according to the National Institutes of Health Guide for the Care and Use of Laboratory Animals and approved by the Institutional Animal Care and Use Committee (IACUC) at University of Florida and Cincinnati Children’s Hospital Medical Center.

### Cell culture and reagents

U2OS or HEK 293 T cells were cultured in Dulbecco’s modified Eagle’s medium (DMEM) supplemented with 10% FBS, 1% L-Glutamine, and 1% penicillin-streptomycin (Invitrogen) at 37 °C under 5% CO2. The cells were transfected with siRNAs using Lipofectamine RNAiMAX (Invitrogen) and DNA plasmids using Fugene HD reagents (Promega). The combined transfection of DNA plasmids with siRNA into the cells were performed using Lipofectamine 2000 (Invitrogen). Cell culture and growth conditions for MMH-D3 hepatocyte were performed as previously described^31^.

### Plasmids

For *NRON* overexpression and generation of stable cell lines, the full-length genomic DNA fragments of *NRON* from U2OS cells was amplified by PCR with primers containing the flanking restriction sites (NotI, XhoI) and inserted into pcDNA3.1 mammalian expression vector (Invitrogen). For plasmids expressing Venus-tagged mouse Per2 (Per2-Venus), full-length DNA fragment of each gene was subcloned into pCMV-Venus, pCMV-VN, or pCMV-VC using a restriction-free (RF) cloning method as described previously^51–53^.

### Antibodies

The following antibodies were used for immunoprecipitation, immunoblotting, and immunostaining: GFP (G1544, Sigma), PER1 (AB2201, Millipore), PER2 (NB100-125, Norvus Biologicals), triphospho-PER2 (Ser662/Ser665/Ser668) (ABN299, Millipore), CRY1 (sc-33177, Santa Cruz Biotech.), CRY2 (13997-1-AP, Proteintech), KPNB1 (A300-482A, Bethyl Lab), IQGAP1 (05-504, Millipore), DYRK1A (ST1650, Calbiochem), PSMD11 (GTX50018, GeneTex), CSNK1E (610445, BD Biosciences), GSK3B (sc-53931, Santa Cruz Biotech), and PPP2R1A (SAB1401297, Sigma), β-Actin (4967, Cell signaling), GAPDH (sc25778, Santa Cruz Biotech), and normal mouse IgG (NI03, Calbiochem).

### Immunofluorescence analysis

After 24 h post-transfection, cells were fixed with 4% paraformaldehyde in PBS and visualized under a fluorescence microscope. For IP analysis, cells were incubated with various antibodies and then secondary antibodies, and visualized using FITC/TRITC/DAPI filter sets. DAPI merged images indicate colocalization. For nuclear and cytoplasmic protein quantification, original red fluorescent images were first converted to white and black mode, nuclear and cytoplasmic areas in each stained cell manually demarcated, and signal intensities measured using Image J software to obtain the ratio.

### RNA-fluorescence *in situ* hybridization (RNA-FISH) analysis

A set of 48 Quasar 570 labeled probes (Stellaris) targeting NRON mRNA were designed using the Stellaris probe designer. RNA-FISH procedure was performed according to the manufacturer’s protocol (Biosearch Technologies). For hybridization, the probes were incubated with control and NRON stable expression cells at 37 °C overnight, followed by immunostaining. Images were acquired using FITC/TRITC/DAPI filter set in fluorescence microscopy.

### RNA-binding protein immunoprecipitation (RIP) assay

Cell lysates were cross-linked by formaldehyde for 15 min and the protein/RNA complex were immunoprecipitated with antibodies. The eluted NRON RNA samples were analyzed RT-PCR to determine the association between RNA and protein of interest.

### Co-immunoprecipitation and immunoblotting

At 48 post-transfection of U2OS cells, cell lysates were harvested in radioimmunoprecipitation assay (RIPA) buffer containing 50 mM HEPES (pH 7.4), 150 mM NaCl, 1% NP-40, 1 mM EDTA, 1 mM EGTA, 1 mM phenylmethylsulfonyl fluoride, 0.5% sodium deoxycholate, 1 mM NaF, 1 mM Na3VO4, and protease inhibitor cocktail (Roche). Protein-G coated magnetic beads (10004D, Life Technologies) were pre-incubated with 3 µg of anti-GFP antibody (G1544, Sigma) at 4 °C for 6 h. Antibody conjugated beads were incubated with equal amounts of total protein at 4 °C overnight. The final immune complexes were analyzed by immunoblot assay.

### RNAi knockdown screening

Human U2OS cells stably expressing the *pBmal*-dLuc construct were reverse transfected with a total of 9.6 nM pooled siRNAs (n = 4/gene) using Lipofectamine/RNAiMAX (Invitrogen) transfection reagent and Opti-MEM media (Gibco), totaling 6 pmol/siRNA for each gene. All siRNAs were purchased from Qiagen. Allstars Negative siRNA (Qiagen) and AllStars Cell Death siRNA (Qiagen) were used as experimental controls. Transfected cells were seeded in triplicate at a density of 200,000 cells/mL in DMEM containing 10% FBS (Gibco), 0.1 mM nonessential amino acids (NEAA, Invitrogen), 1% L-Glutamine (Invitrogen) without antibiotics into 96-well plates and incubated for 24 h. siRNA/transfection medium was removed 24 h post-transfection and replaced with 250 µl of luminescence recording medium: phenol red-free DMEM (Sigma D-2902), sodium bicarbonate (Invitrogen), D-(+)-glucose (Sigma), 10 mM HEPES (Invitrogen), 1% pen/strep/L-glutamine (Invitrogen), and 0.1 mM luciferin (Promega). The medium is supplemented with 0.1 µM dexamethasone (Sigma-Aldrich) to synchronize the cells to the same circadian phase. The Synergy 2 plate reader (BioTek) was used to measure luminescence every 1 h for 6 days. Period length was determined using the WaveClock algorithm implemented in R.

### Transfection efficiency validation for RNAi knockdown screen

To validate the siRNA knockdown efficiency, cells were synchronized and transferred to luminescence recording medium as described above in parallel to the luminescence experiment and harvested 48 h post transfection. RNA was extracted using the Direct-zol-96 RNA kit (Zymo Research). qScript RT-transcriptase (Quanta) was used for reverse transcription reactions. Quantitative PCR was performed using PerfeCTa FastMix II, Low ROX (Quanta). All Taqman primers were purchased from Applied Biosystems.

### Cell viability assay

The ATP-lite Luminescence ATP Detection Assay System (PerkinElmer) was used to determine cell viability 96 h post-transfection of selected siRNAs from Fig. 1 (n = 6 replicates per time point). Briefly, human U2OS cells stably expressing the *pBmal1*-dLuc construct were reverse transfected with a total of 9.6 nM pooled siRNAs (n = 4/gene) as above using Lipofectamine/RNAiMAX (Invitrogen) transfection reagent and Opti-MEM media (Gibco), totaling 6 pmol/siRNA for each gene. All siRNAs were purchased from Qiagen. Allstars Negative siRNA (Qiagen), Lipofectamine (Invitrogen) only, and untreated cells were used as negative controls and AllStars Cell Death siRNA (Qiagen) was used as a positive control. *BMAL1*, *CRY1* and *CRY2* were used as molecular clock phenotype controls. Transfected cells were seeded (n = 6 per treatment) at a density of 12,000 cells/well to be within the linear range of the assay in DMEM containing 10% FBS (Gibco), 0.1 mM nonessential amino acids (NEAA, Invitrogen), 1% L-Glutamine (Invitrogen) without antibiotics into 96-well plates and incubated for 24 h. siRNA/transfection medium was removed 24 h post-transfection and replaced with 100 µl of luminescence recording medium without luciferin: phenol red-free DMEM (Sigma D-2902), sodium bicarbonate (Invitrogen), D-(+)-glucose (Sigma), 10 mM HEPES (Invitrogen), and 1% pen/strep/L-glutamine (Invitrogen). The assay was done following the manufacturer’s protocol (PerkinElmer). Luminescence was detected in the Synergy Neo2 microplate reader (BioTek) 96 h post-transfection. The mean luminescence data (counts/s) were plotted using ggplot2 in R (n = 6 replicates).

### SCN dissection and real-time bioluminescence recording

SCN and peripheral tissue slices were dissected and cultured in explant medium as described previously^31,41^. For real-time bioluminescence recording, we used a Lumicycle luminometer (Actimetrics) on 35-mm culture dishes. Lumicycle Analysis Program (Actimetrics) was used to analyze Lumicycle data to determine period length and rhythm amplitude. Briefly, raw data were fitted to a linear baseline, and the baseline-subtracted data were fitted to a sine wave (damped), from which period length and goodness of fit and damping constant were determined. For samples that showed persistent rhythms, goodness-of-fit of >80% was usually achieved. Due to high transient luminescence upon medium change, the first cycle was usually excluded from rhythm analysis. For amplitude analysis, raw data from day 3 to day 5 were fitted to a linear baseline, and the baseline-subtracted (polynomial number = 1) data were fitted to a sine wave, from which the amplitude was determined.

### Statistical analysis

All statistical tests were done using Excel or Prism7 GraphPad Software. For making multiple comparisons, we used one way ANOVA followed by Dunnett’s multiple-comparisons test or the Student’s t-test (two-tailed paired or unpaired) to reject the null hypothesis (p < 0.05). For bioluminescence rhythm data from U2OS and SCN explants, data are represented as mean +/− SD and p-value was determined by two-tailed t-test.

## Supplementary information

Supporting information

## Data Availability

The datasets generated during and/or analyzed during the current study are available from the corresponding author on reasonable request.
