# Supplementary material for: The NRON complex controls circadian clock function through regulated PER and CRY nuclear translocation
Source: Sci Rep. 2019 Aug 15;9:11883. doi: 10.1038/s41598-019-48341-8 (PMC6695496; doi:10.1038/s41598-019-48341-8)
Supplement: Supplementary file 1 — Supporting information [file 41598_2019_48341_MOESM1_ESM.pdf]

Supporting information for:

The NRON complex controls circadian clock function through regulated PER and CRY nuclear translocation

Yool Lee[1,2], Yang Shen[3,4], Lauren J. Francey[1,5], Chidambaram Ramanathan[4], Amita Sehgal[2], Andrew C. Liu[3,4], John B. Hogenesch[1,5*]

[1] Department of Systems Pharmacology and Translational Therapeutics, University of Pennsylvania Perelman School of Medicine, Philadelphia, PA 19104

[2] Penn Chronobiology, Howard Hughes Medical Institute, Dept of Neuroscience, Perelman School of Medicine, University of Pennsylvania, Philadelphia, PA. 19104

[3] Department of Physiology and Functional Genomics, University of Florida College of Medicine, Gainesville, FL 32610

[4] Department of Biological Sciences, University of Memphis, Memphis, TN 38152

[5] Divisions of Human Genetics and Immunobiology, Cincinnati Children's Hospital Medical Center, Cincinnati, OH 45229

*Correspondence to: John Hogenesch (john.hogenesch@cchmc.org)

Figure S1

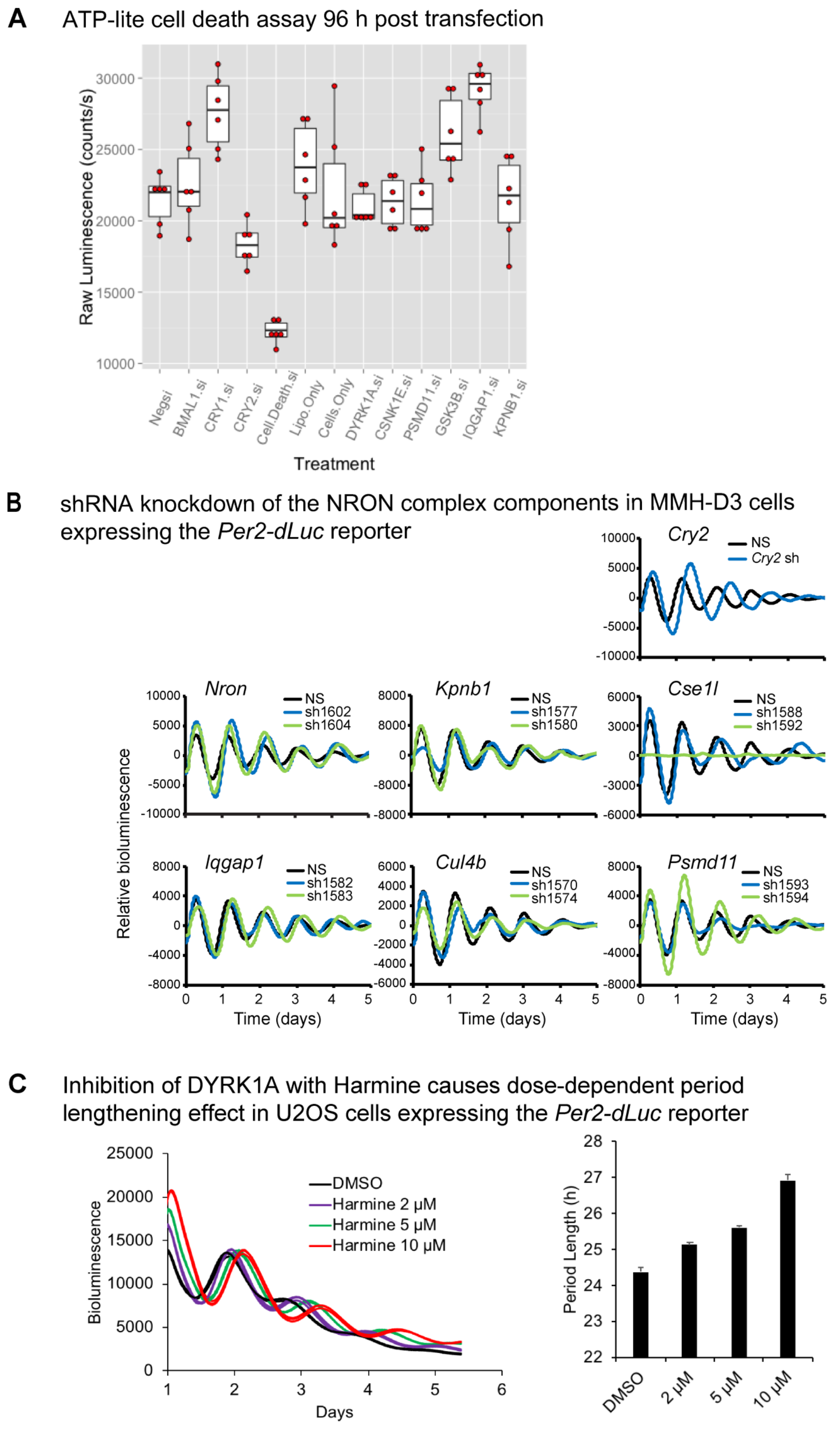

Figure S1. Effects of RNAi knockdown of the NRON complex components on circadian oscillations in cellular clock models. **(A)** Cytotoxicity of selected siRNA pools using the ATP-lite cell viability assay 96 h post-

transfection (n= 6 replicates per treatment). The Cell Death siRNA (Cell.Death.si) pool had ~50% cell viability compared to the negative siRNA control (Negsi) at this time point. **(B)** shRNA knockdown effects of the NRON complex components in MMH-D3 hepatocyte cells expressing the *Per2-dLuc* reporter. *Nron, Kpnb1, Cse1l, Iqgap1, Cul4b* and *Psmd11* are the NRON complex components. NS (non-specific), negative control. *Cry2*, positive control. **(C)** Pharmacological inhibition of DRYK1A lengthens period length in U2OS cells expressing the *Per2-dLuc* reporter. Representative bioluminescence records (left) and quantified period length (right) are shown for DMSO and different dosages of Harmine.

Figure S2

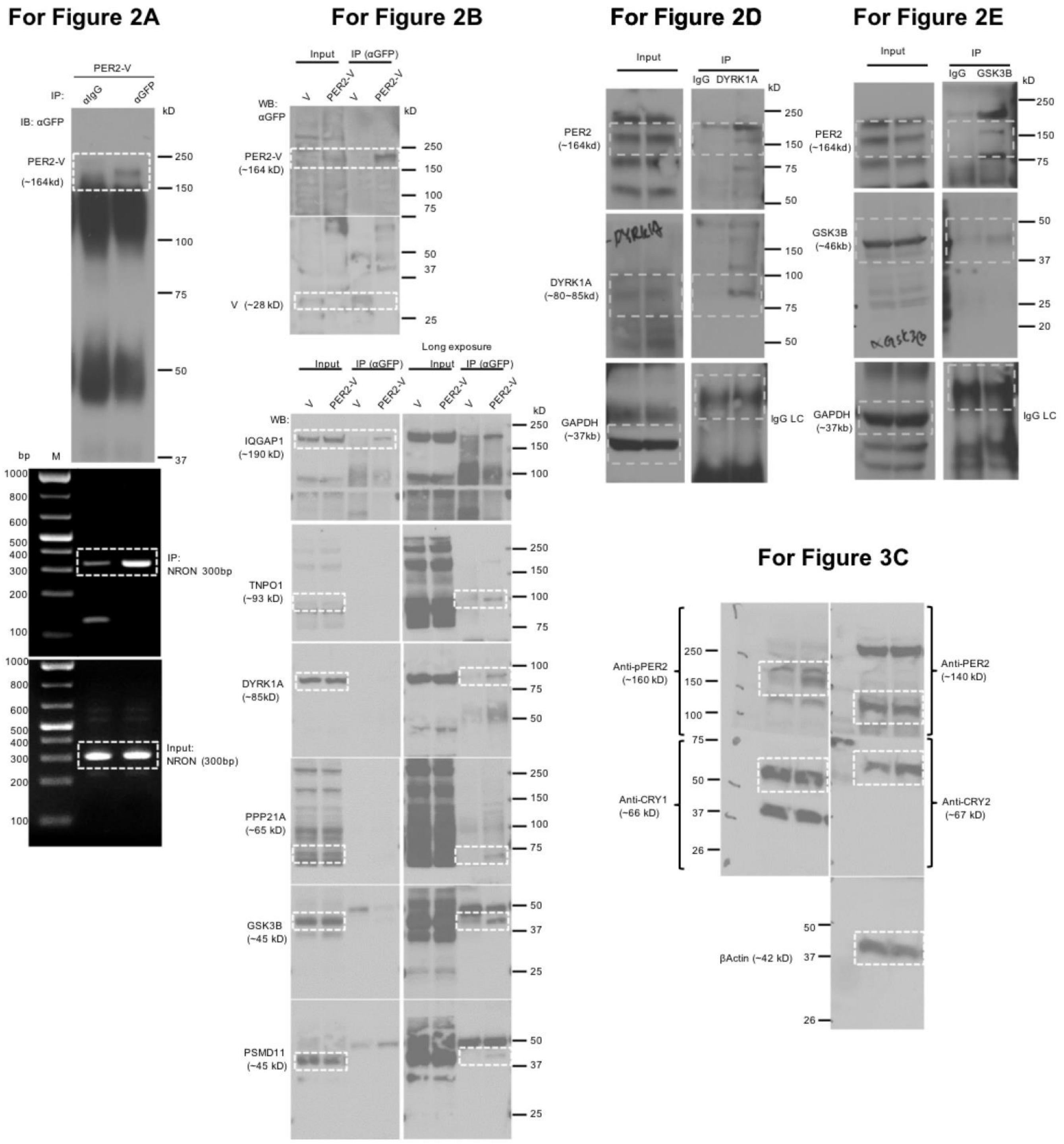

Figure S2. Original blot data for Figure 2 and Figure 3C. The clipped blot images are indicated with white dashed rectangles.

Figure S3

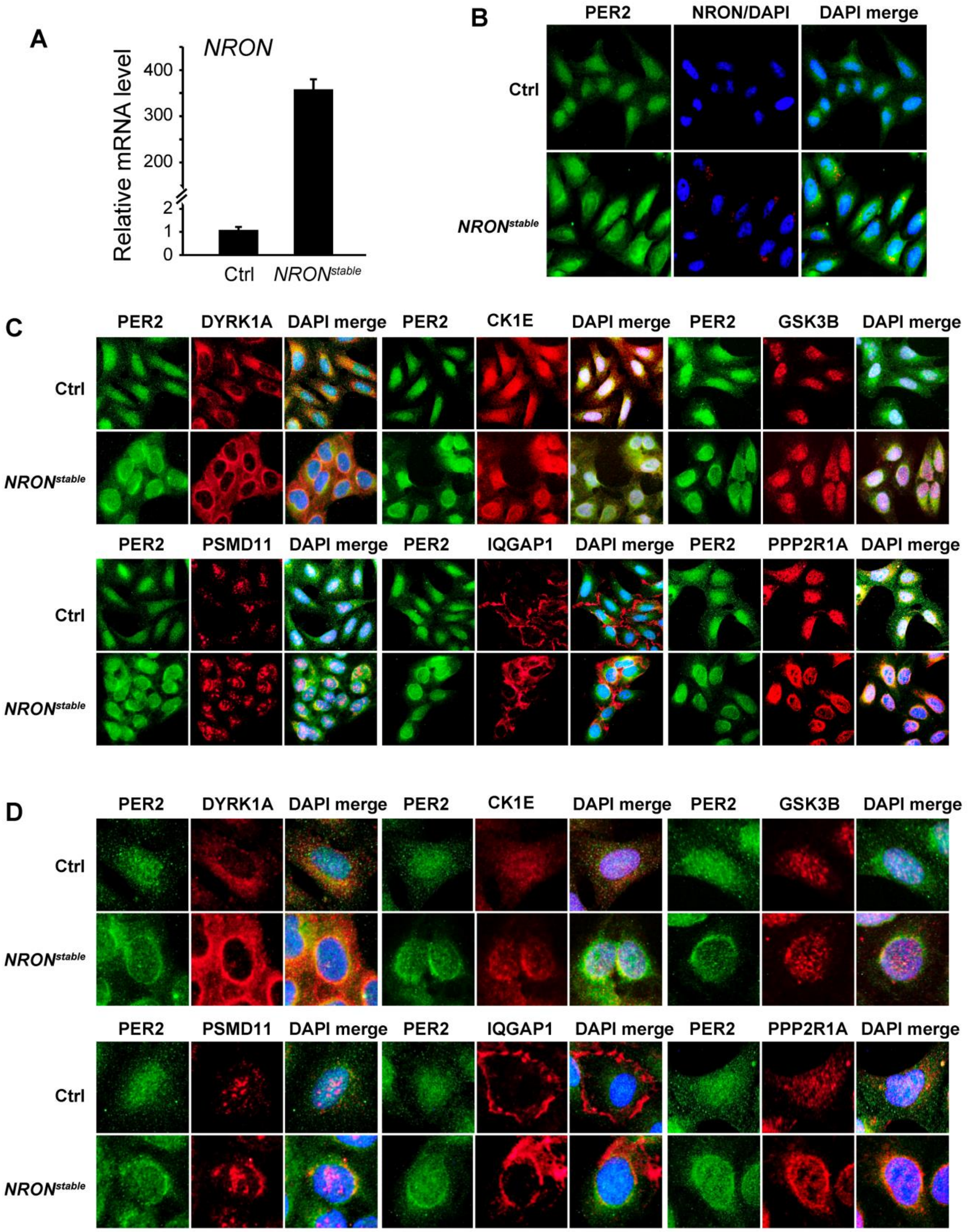

Figure S3. PER2 colocalizes with NRON complex components at the perinuclear region. **(A)** Quantitative RT-PCR analysis of NRON expression in control U2OS cells (Ctrl) and cells stably overexpressing *NRON* (*NRON*^{Stable}). Data are mean ± SE of triplicate samples. **(B)** Combined RNA fluorescence in situ hybridization (RNA FISH) and immunofluorescence analysis of subcellular localization of *NRON* and PER2. Fluorescence microscopy filter sets: FITC (green, PER), TRITC (red, *NRON*), and DAPI (blue, nucleus). **(C)** Immunofluorescence analysis of subcellular localization of PER2 and NRON complex components.

Fluorescence microscopy filter sets: FITC (green, PER2), TRITC (red, NRON complex components as indicated), and DAPI (blue; nucleus). **(D)** Representative single cell images from (C).

Figure S4

A siRNA knockdown of the catalytic subunits of calcineurin (*PPP3CA, CB and CC*) in human U2OS cells expressing the *Per2-dLuc* reporter

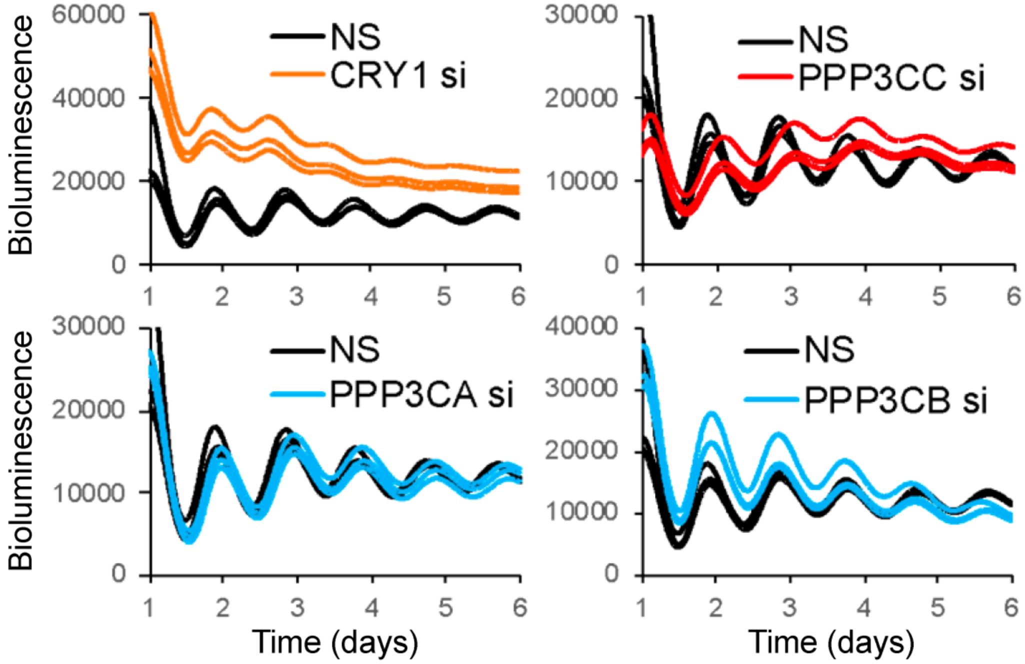

B shRNA knockdown of the catalytic subunit of calcineurin (*Ppp3cc*) in mouse MMH-D3 cells expressing the *Per2-dLuc* reporter

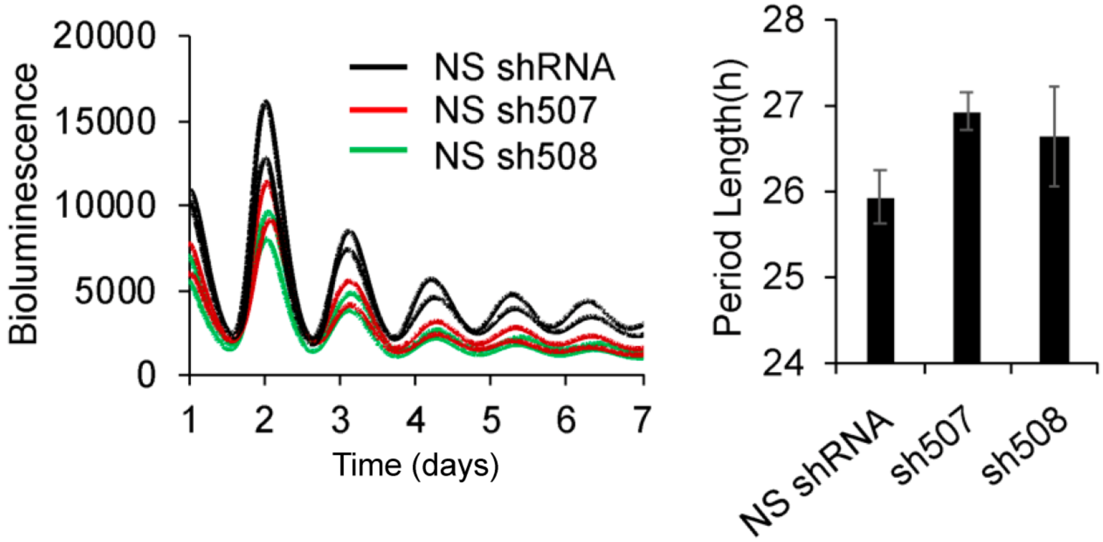

Figure S4. Effects of knockdown of the catalytic subunit (*PPP3CC*) of calcineurin (CaN) on period length. **(A)** siRNA knockdown of the catalytic subunits of CaN (*PPP3CA, PPP3CB* and *PPP3CC*) in human U2OS cell expressing the *Per2-dLuc* reporter. NS, non-specific. *CRY1* serves as a positive control. **(B)** Lentiviral shRNA knockdown of *Ppp3cc* caused long period length in mouse MMH-D3 cells expressing the *Per2-dLuc* reporter.

Two independent shRNA constructs against *Ppp3cc* were used. NS, non-specific. Representative bioluminescence records (left) and quantified period length data (right) are shown.

Figure S5

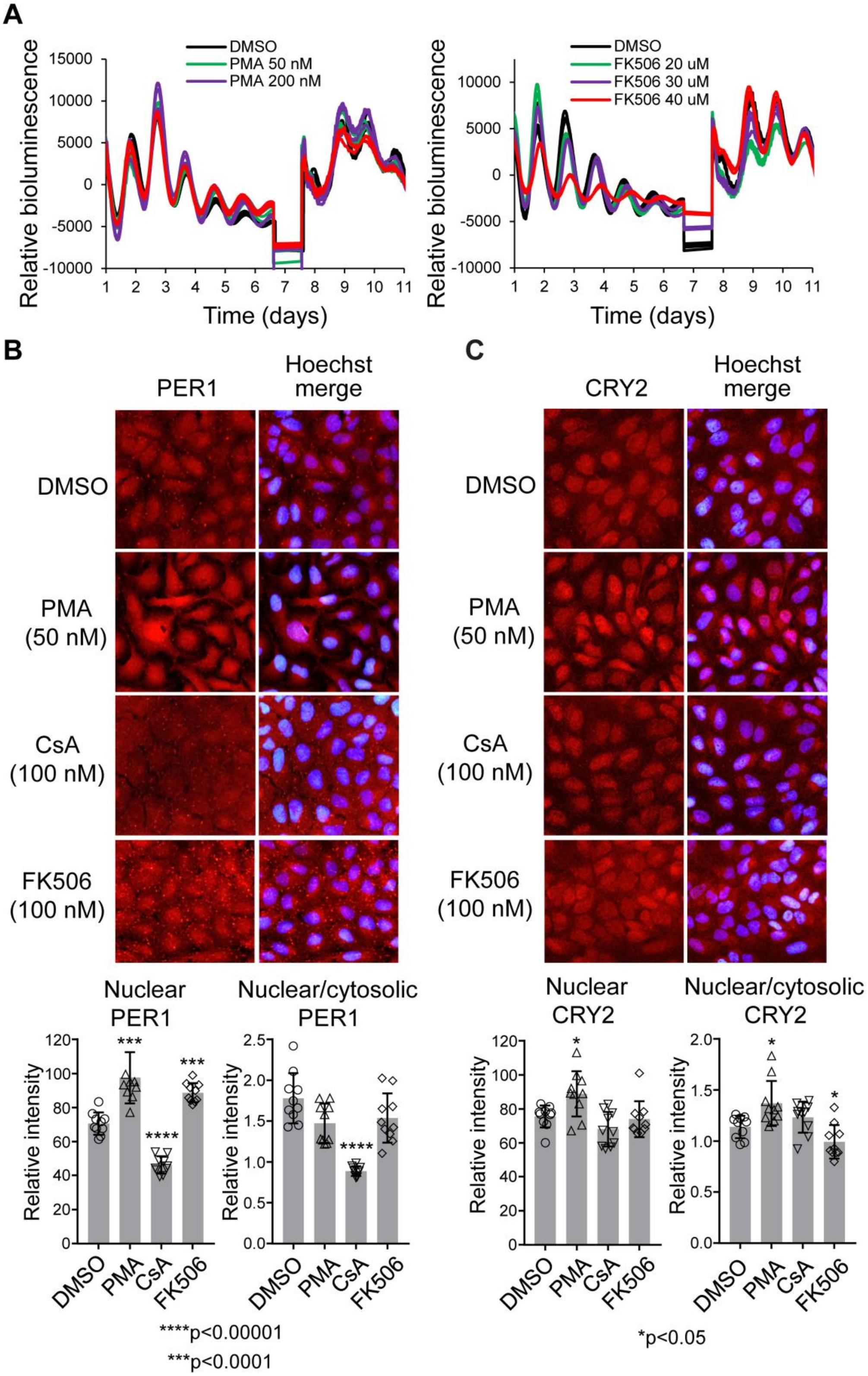

Figure S5. Effects of pharmacological perturbation of the NRON/NFAT pathway on circadian rhythms and nuclear localization of PER1 and CRY2 in U2 OS cells. PMA, activator. CsA and FK506, inhibitors. **(A)** Representative bioluminescence records of circadian rhythms in U2OS cells expressing the *Bmal1-Luc*

reporter upon PMA (left) or FK506 (right) treatment. Cellular toxicity was not shown in varying doses of PMA (50~200 nM) and FK506 (20~40 μM) as bioluminescence rhythms of the the drug-treated cells were reactivated as robustly as DMSO-treated controls when resynchronized. (**B-C**) PER1 and CRY2 proteins were detected by immunostaining using anti-PER1 and anti-CRY2 antibodies. Representative images (upper) are shown with quantitative assessment of nuclear/cytoplasmic ratio (lower) for the effect on PER1 and CRY2 nuclear abundance after 6 h of treatment. Data are presented as mean ± SD (n = 10); *p<0.05, ***p<0.00001 by two-tailed student's t-test.
